# Efficacy and safety of various surgical treatments for proximal ureteral stone ≥10mm: A systematic review and network meta-analysis

**DOI:** 10.1590/S1677-5538.IBJU.2019.0550

**Published:** 2020-09-02

**Authors:** Yaxuan Wang, Xueliang Chang, Jingdong Li, Zhenwei Han

**Affiliations:** 1 Department of Urology The Second Hospital Hebei Medical University Shijiazhuang China Department of Urology, The Second Hospital of Hebei Medical University, Shijiazhuang, China

**Keywords:** Lithotripsy, Ureteroscopy, Nephrolithotomy, Percutaneous

## Abstract

**Purpose:**

Various surgical options are available for large proximal ureteral stones, such as extracorporeal shock wave lithotripsy (ESWL), ureteroscopic lithotripsy (URSL), percutaneous nephrolithotomy (PCNL) and laparoscopic ureterolithotomy (LU). However, the best option remains controversial. Therefore, we conducted a network meta-analysis comparing various surgical treatments for proximal ureteral stones ≥10mm to address current research deficiencies.

**Materials and methods:**

We searched PubMed, Ovid, Scopus (up to June 2019), as well as citation lists to identify eligible comparative studies. All clinical studies including patients comparing surgical treatments for proximal ureteral stones ≥10mm were included. A standard network meta-analysis was performed with Stata SE 14 (Stata Corp, College Station, TX, USA) software to generate comparative statistics. The quality was assessed with level of evidence according to the Oxford Centre for Evidence-based Medicine and risk of bias with the Cochrane Collaboration’s Review Manager (RevMan) 5.3 software.

**Results:**

A total of 25 studies including 2.888 patients were included in this network meta-analysis. Network meta-analyses indicated that LU and PCNL had better stone-free rates and auxiliary procedures. PCNL could result in major complications and severe bleeding. In initial stone-free rate, final stone-free rate, and auxiliary procedures results, SUCRA ranking was: LU> PCNL> URSL> ESWL. In Clavien Dindo score ≥3 complications, SUCRA ranking was: LU> ESWL> URSL> PCNL. In fever, SUCRA ranking was: ESWL> LU> URSL> PCNL. In transfusion, SUCRA ranking was: LU> URSL> ESWL> PCNL. In Cluster analysis, LU had the highest advantages and acceptable side effects. Considering the traumatic nature of PCNL, it should not be an option over URSL. ESWL had the lowest advantages.

**Conclusions:**

LU have the potential to be considered as the first treatment choice of proximal ureteral stone ≥10mm.

## INTRODUCTION

Urolithiasis is one of the most common health care burdens in the daily lives of working-age people ( 1 ). Ureteral stones with a diameter of less than 6mm are generally considered to be associated with spontaneous passage, while stones with a diameter of more than 10mm are less likely to pass spontaneously ( 2 ). So, large ureteral stones above 10mm require further intervention. Due to the long distance, the proximal ureteral stones are not easy to pass, and it is easier to form a stone street. In addition, approaching proximal ureter and stone migration are two major challenges for ureteroscopy. Therefore, the treatment of large proximal ureteral stones is more difficult. With the development of medical equipment and improved skills, various techniques can be used to treat large ureteral stones, especially the proximal ureteral stones. Among various treatments of proximal ureteral stones, such as extracorporeal shock wave lithotripsy (ESWL), ureteroscopic lithotripsy (URSL), percutaneous nephrolithotomy (PCNL) and laparoscopic ureterolithotomy (LU), the best choice remains controversial ( 3 - 5 ). According to the EAU Guidelines, ESWL remains the first line treatment modality for ureteral stones less than 2cm, because of its non-invasive nature ( 6 ). However, large impacted proximal ureteral stones could be related with lower stone-free rate. URSL has been increasingly used to treat proximal ureteral stones. Due to the risk of stone migration, there is still a debate on its efficacy ( 7 ). It has been reported that both PCNL and LU have higher efficacy despite the more complicated surgical procedures and more complications ( 8 , 9 ).

A number of studies have investigated the efficacy and safety of different surgical treatments for large proximal ureteral stones. However, the best way to treat the large proximal ureteral stones remains to be determined. Therefore, we performed a network meta-analysis to compare the stone-free rate and complications of various surgical treatments of large proximal ureteral stones.

## MATERIALS AND METHODS

### Literature search

We performed a systematic review up to June 2019 in accordance with the Preferred Reporting Items for Systematic Reviews and Meta-Analysis Statement. Research papers from PubMed, Ovid and Scopus databases were searched to identify eligible studies. The search strategy was “(proximal ureteral stone OR proximal ureteral calculi OR upper ureteral stone OR upper ureteral calculi OR upper ureterolithiasis) AND (extracorporeal shock wave lithotripsy OR ESWL OR ureteroscopy OR ureterolithotripsy OR ureterolithotomy OR laparoscopy OR laparoscopic ureterolithotomy OR percutaneous nephrolithotomy OR PCNL OR surgery)”.

### Inclusion and exclusion criteria

Inclusion criteria: ( 1 ) original studies comparing different surgical treatments for proximal ureteral stones; ( 2 ) proximal ureteral stones ≥10mm; ( 3 ) studies reported in English language; ( 4 ) adult patients only; ( 5 ) the outcomes should include stone-free rate, auxiliary procedures, transfusion, fever and other complications. Exclusion criteria: ( 1 ) studies without primary data, such as reviews, commentaries, conference abstracts; ( 2 ) duplicated publications; ( 3 ) no sufficient data; ( 4 ) combined with middle or distal ureteral stones; ( 5 ) previously failed interventions or combined with infections. These studies were performed in compliance with the Preferred Reporting Items for Systematic Reviews and Meta-Analyses (PRISMA) statement. (Supplementary Table-1).

### Data extraction

Two authors (YW and XC) independently extracted data using a predefined standard data extraction form. Any discrepancy was resolved by discussion with a third reviewer (JL). The following data were extracted: baseline demographics (age, gender and stone size), primary outcomes (initial and final stone-free rate) and secondary outcomes (auxiliary procedures, fever, transfusion and Clavien Dindo score ≥3 complications). The initial stone-free rate was defined as stone-free rate after first procedure, while the final stone-free rate was defined as stone-free rate after final procedure. The surgical treatments in this study included extracorporeal shock wave lithotripsy (ESWL), ureteroscopic lithotripsy (URSL), ureteroscopic lithotripsy-retrograde intrarenal surgery (URSL-RIRS), percutaneous nephrolithotomy (PCNL), mini-percutaneous nephrolithotomy (mPCNL), and laparoscopic ureterolithotomy (LU). There were not enough studies about URSL-RIRS, and mPCNL In addition, due to similar risk of complications and surgical outcomes, we combined mPCNL with PCNL, and URSL-RIRS with URSL.

### Risk of bias evaluation

The Cochrane Collaboration’s Risk of Bias tool was used to evaluate the quality of each study ( 10 ). It includes seven domains: random sequence generation, allocation concealment, blinding of participants, blinding of outcome assessment, incomplete outcome data, selective reporting and other bias. The risk of bias graph and risk of bias summary were conducted using Cochrane Collaboration’s Review Manager (RevMan) 5.3 software (Cochrane Collaboration, Oxford, UK).

### Quality assessment

The quality of included studies was assessed by level of evidence according to the Oxford Centre for Evidence-based Medicine.

### Statistical analysis

A Bayesian network meta-analysis was performed to compare different surgical treatments with each other using Stata SE 14 (Stata Corp, College Station, TX, USA). A standard network model was established and the OR with 95% confidence intervals (CIs) of each parameter were worked out. Network forest plots and loop inconsistency test were employed to determine the global consistency. In addition, the node-splitting method was used to identify the consistency between direct and indirect evidences. When the node-splitting results were p >0.05, the consistency model was executed. The surface under the cumulative ranking (SUCRA) was used to assess the probability that each intervention is the most effective or safest surgical treatment based on Bayesian approach. The larger the SUCRA value, the greater the probability of being effective ( 11 ). Cluster analysis was applied on the SUCRA scores to evaluate the efficacy and tolerability. Networ funnel plots were examined to evaluate publication bias.

## RESULTS

**Figure 1:**
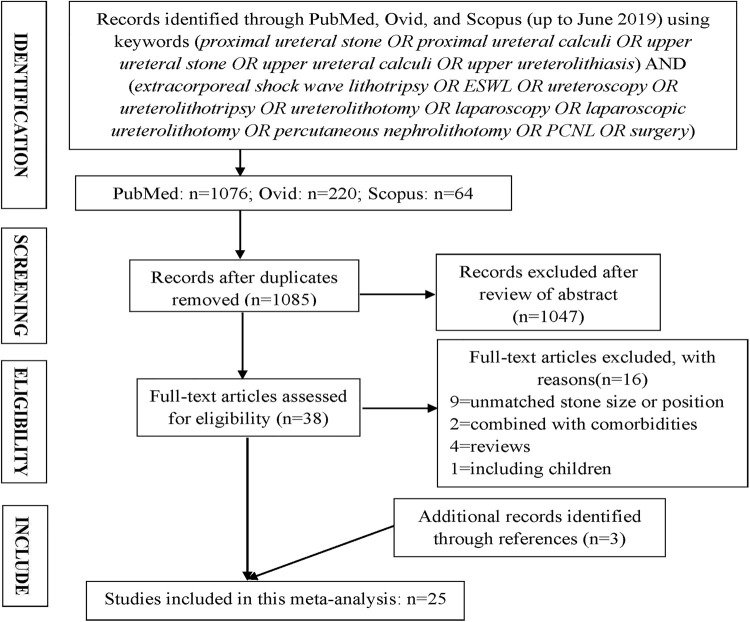
PRISMA flow diagram detailing the search strategy and identification of studies included in data synthesis.

**Figure 2:**
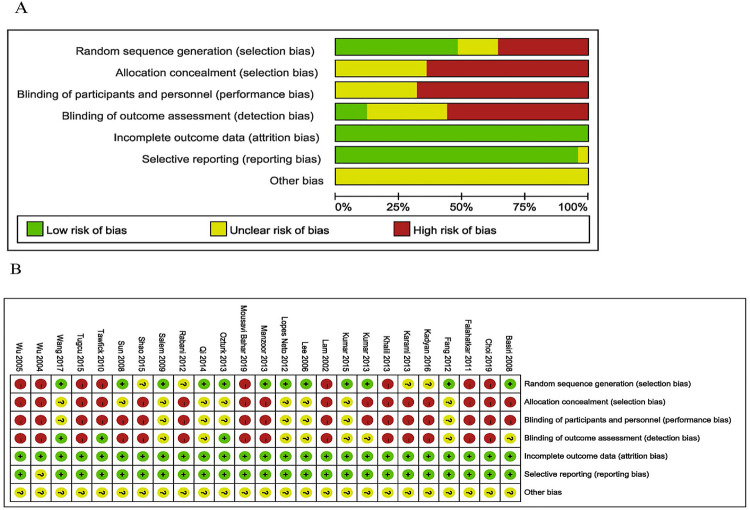
A) Risk of bias graph, review authors´ judgements about each risk of bias item presented as percentages. B) Risk of bias summary, review authors´ judgements about each ris of bias item for each included study.

Overall, 25 studies including 2.888 patients were included in this network meta-analysis ( Figure-1 ) ( 12 - 36 ). The baseline characteristics and the risk of bias for the included 25 studies are displayed in Table-1 and Figure-2 , respectively.

**Table 1:** Characteristics of the enrolled studies for this meta-analysis.

Category	Study	Study design	LE	Study region	Follow-up time	Definition of stone-free	Methods	Gender (male, n)	Age (years)	Stone size (mm)
ESWL vs URSL	Khalil, et al. 2013 ( 12 )	n-RCT	4	Kuwait	3 months	Complete removal	ESWL	31, 37	37.1±8.8	13.2±2.9
							URSL	37, 45	35.2±10.4	13.4±2.7
	Lee, et al. 2006 ( 13 )	RCT	2b	China	Final procedures	≤ 3 mm	ESWL	19, 22	54.2±16.7	17.9±3.9
							URSL	16, 20	48.5±13.3	18.5±2.9
	Salem, et al. 2009 ( 14 )	RCT	2b	Egypt	3 months	Complete removal	ESWL	27, 42	36.4±4.5	12.5±2.3
							URSL	30, 48	36.7±7	12.2±2
	Kumar, et al. 2013 ( 15 )	RCT	2b	India	3 months	≤ 3 mm	ESWL	20, 37	37.3±2.2	15.2±1.3
							URSL	21, 41	36.3±2.3	15.3±1.2
	Manzoor, et al. 2013 ( 16 )	RCT	2b	Pakistan	Not stated	Not stated	ESWL	NA	44.3±10.1	10.8±4.3
							URSL	NA	45.4±13.2	11.3±3.7
	Tawfick, et al. 2010 ( 17 )	n-RCT	4	Egypt	1 month	Not stated	ESWL	54, 71	NA	13.4±0.3
							URSL	61, 76	NA	15.1±0.4
	Wu, et al. 2004 ( 18 )	n-RCT	4	China	1 month	Not stated	ESWL	34, 41	NA	12.8±0.4
							URSL	34, 39	NA	15.1±0.5
	Wu, et al. 2005 ( 19 )	n-RCT	4	China	4 weeks	< 3 mm	ESWL	41, 51	51.5±1.9	12.1±0.3
							URSL	43, 56	53.8±1.5	17±0.7
	Lam, et al. 2002 ( 20 )	n-RCT	4	USA	3 months	Complete removal	ESWL	14, 20	45.4±5	12.6±2.5
							URSL	12, 14	39.6±7	11.1±2.5
	Rabani, et al. 2012 ( 21 )	RCT	2b	Iran	1 month	< 5 mm	ESWL	NA	NA	17.7±3.3
							URSL	NA	NA	17.6±3.8
URSL vs PCNL	Qi, et al. 2014 ( 22 )	RCT	2b	China	1 month	< 4 mm	URSL	31, 52	42.5±10.3	19.8±4.3
							PCNL	30, 52	41.1±12.4	20.3±3.6
	Sun 2008, et al. ( 23 )	RCT	2b	China	1 month	< 5 mm	URSL	31, 47	39.6±7.3	14.6±1.8
							PCNL	30, 44	40.4±8.4	14.7±2
URSL vs LU	Fang, et al. 2012 ( 24 )	RCT	2b	China	3-12 months	Not stated	URSL	15, 25	36.9±11.8	15±4
							LU	14, 25	34.4±9.8	16±3
	Kumar, et al. 2015 ( 25 )	RCT	2b	India	3 months	≤ 3 mm	URSL	26, 50	35.6±2.1	22±1
							LU	24, 50	36.7±2.4	23±2
	Shao, et al. 2015 ( 26 )	RCT	2b	China	20 months	Not stated	URSL	90, 139	41±12.3	13.6±1.4
							LU	92, 136	40±12.5	13.8±1.9
URSL vs LU	Choi, et al. 2019 ( 27 )	n-RCT	4	South Korea	3 months	< 2 mm	URSL	32, 52	57±1.5	2.2±0
							LU	26, 48	57.9±1.9	2.1±0
	Falahatkar, et al. 2011 ( 28 )	n-RCT	4	Iran	Not stated	Not stated	URSL	12, 20	43±14	NA
							LU	14, 20	41±10	NA
	Kadyan, et al. 2016 ( 29 )	RCT	2b	India	3 weeks	< 4 mm	URSL	38, 60	44.3±3.2	16.8±1.5
							LU	37, 62	42.1±2.7	17.2±1.9
	Tugcu, et al. 2016 ( 30 )	n-RCT	4	Turkey	1 month	< 4 mm	URSL	55, 80	40.7±10.2	18.5±3.4
							LU	73, 103	39.9±12	21.1±4.5
PCNL vs LU	Karami, et al. 2013 ( 31 )	RCT	2b	Iran	6 months	Complete removal	PCNL	28, 40	39.4±11.8	14.2±3.8
							LU	24, 40	35.2±9.8	13.5±4.5
	Mousavi, et al. 2019 ( 32 )	n-RCT	4	Iran	Not stated	Not stated	PCNL	39, 52	47.8±16.7	18.3±2.6
							LU	46, 55	42.9±16.1	21.3±2.2
ESWL vs URSL vs LU	Lopes Neto, et al. 2012 ( 33 )	RCT	2b	Brazil	2 months	≤ 3 mm	ESWL	7, 14	46±13.5	13.8±2.5
							URSL	10, 16	49.6±15.5	14.4±4.1
							LU	9, 15	46±13.6	15.9±4.1
	Ozturk, et al. 2013 ( 34 )	RCT	2b	Turkey	3 months	< 4 mm	ESWL	33, 52	40.7±14.5	13.2±2.1
							URSL	30, 48	41.1±8.5	13.2±2
							LU	21, 51	40±10.8	13.3±2.1
URSL vs PCNL vs LU	Basiri, et al. 2008 ( 35 )	RCT	2b	Iran	3 weeks	Not Stated	URSL	33, 50	39±15	17.8±2.4
							PCNL	32, 50	48±13	20.3±3.3
							LU	36, 50	44±13	22.4±3.2
	Wang, et al. 2017 ( 36 )	RCT	2b	China	1 month	< 4 mm	URSL	28, 50	42±14	16.8±2.1
							PCNL	31, 50	41±15	19.3±1.8
							LU	29, 50	44±11	18.8±1.4

**n=** number; mm= millimeter; **n-RCT** = non-randomized controlled trial; **RCT** = randomized controlled trial; **LE** = level of evidence; **NA** = not available.

**Figure 3:**
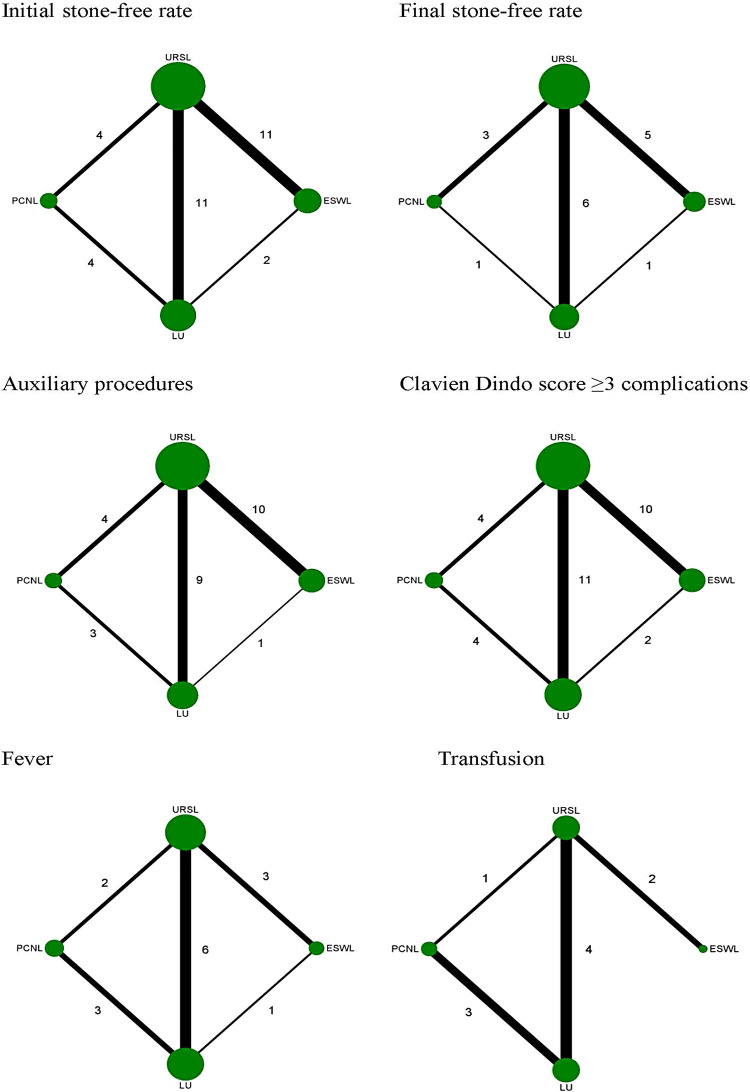
Network maps of included studies fo initial stone-free rate, final stone-free rate, auxiliary procedures, Clavien Dindo score ≥ 3 complications, fever and trandfusion.

There were sixteen RCT studies ( 13 - 16 , 21 - 26 , 29 , 31 , 33 - 36 ) and nine non-RCT studies ( 12 , 17 - 20 , 27 , 28 , 30 , 32 ) included in this study ( Table-1 ). Twenty three studies reported URSL, making it the most commonly used treatment ( 12 - 30 , 33 - 36 ). Thirteen studies reported LU ( 24 - 36 ), twelve studies reported ESWL ( 21 - 21 , 33 , 34 ), and six studies reported PCNL ( 22 , 23 , 31 , 32 , 35 , 36 ). Twenty four studies reported initial stone-free rate ( 12 - 14 , 16 - 36 ). Twelve studies reported final stone-free rate ( 12 , 19 - 27 , 33 , 35 ). Twenty one studies reported auxiliary procedures ( 12 - 15 , 17 - 27 , 29 , 30 , 32 , 33 , 35 , 36 ). Twenty three studies reported Clavien Dindo score ≥3 complications ( 12 , 13 , 15 , 17 - 36 ). Eleven studies reported fever ( 12 , 13 , 22 , 26 - 28 , 30 - 32 , 34 , 36 ). Eight studies reported transfusion ( 15 , 21 , 27 , 28 , 30 - 32 , 36 ). The network maps of the included studies reported the primary outcomes (initial and final stone-free rate) and secondary outcomes (auxiliary procedures, fever, transfusion and Clavien Dindo score ≥3 complications) are shown in Figure-3 .

The results showed no significant difference in terms of initial stone-free rate, final stone-free rate, auxiliary procedures, Clavien Dindo score ≥3 complications, fever and transfusion (all p >0.05) (Supplementary Figure-1). The node-splitting results showed consistency between all the direct and indirect evidences (all p >0.05) ( Table-2 ). The loop inconsistency test results showed that all direct and indirect evidences were consistent in each parameter. So, the consistency model was used for further analysis (all 95% CIs including 0) (Supplementary Figure-2).

**Table 2:** Node-splitting results of the four treatments under the six endpoint outcomes.

Pairwise comparisons	Direct OR values							Indirect OR values							P values					
		
	iSFR		fSFR	AP	C3	F	T	iSFR	fSFR	AP	C3	F	T		iSFR	fSFR	AP	C3	F	T
ESWL vs URSL	0.95	0.46	-0.66	0.50	1.37	-0.02		0.15	3.24	0.24	1.03	3.44	-0.84		0.66	0.24	0.75	0.81	0.38	1.00
ESWL vs LU	2.21	3.15	-2.51	-0.00	1.96	NA		3.24	1.33	-2.62	-0.06	0.68	NA		0.29	0.13	0.95	0.97	0.46	NA
URSL vs PCNL	1.39	0.96	-1.46	0.69	-0.22	1.94		1.23	1.00	-1.58	0.31	0.36	1.85		0.84	1.00	0.95	0.78	0.56	0.96
URSL vs LU	2.10	1.09	-1.92	-0.49	-0.36	-0.03		1.48	3.32	-2.43	-1.38	-1.19	0.16		0.56	0.09	0.76	0.51	0.43	0.96
PCNL vs LU	1.05	0.43	-0.62	-1.11	-0.70	-1.90		0.20	-0.32	-0.27	-1.06	0.01	-1.98		0.43	0.50	0.82	0.97	0.50	0.99

**OR** = odds ratios; **NA** = not available; **iSFR** = initial stone-free rate; **fSFR** = final stone-free rate; **AP** = auxiliary procedures; **C3** = Clavien Dindo score ≥3 complications; **F** = fever; **T** = transfusion.

**Figure 4:**
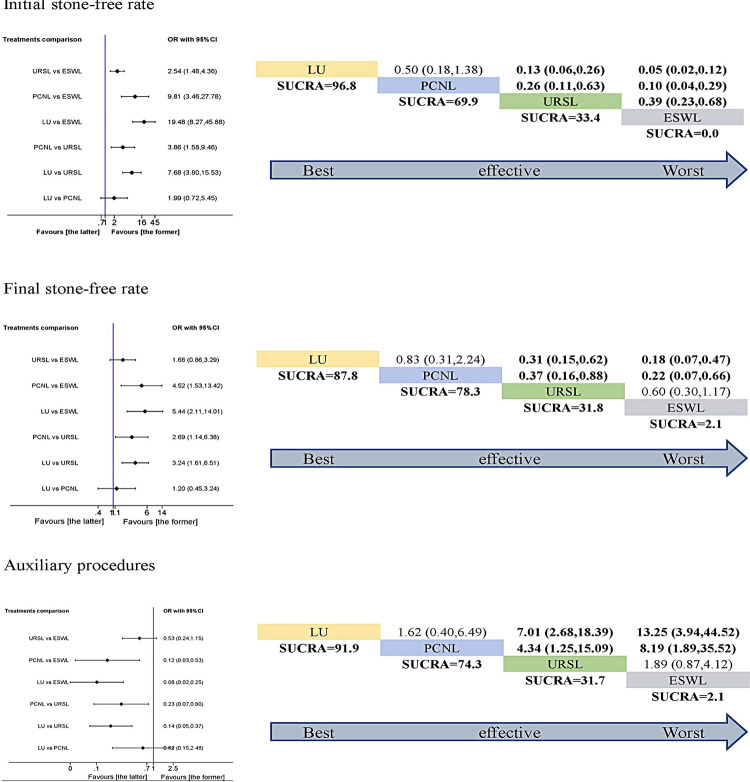
Pairwise meta-analysis (left) and SUCRA rank (right) in terms of initial stone-free rate, final stone-free rate and auxiliary procedures. If the 95% CI was above or under 1.00, the difference was statistically significant (P< 0.05).

The network meta-analysis and SUCRA rank were performed in the six parameters. For initial stone-free rate, LU had the highest SUCRA score, followed by PCNL. While, ESWL had the lowest SUCRA score. Both LU and PCNL were more effective than URSL or ESWL, and URSL was more effective than ESWL (p <0.05). However, the difference between LU and PCNL was not statistically significant (p >0.05). So, we could not draw the conclusion that LU was more effective than PCNL. The SUCRA outcome for initial stone-free rate indicated the following ranking: LU> PCNL> URSL> ESWL. For final stone-free rate, the SUCRA rank was the same as initial stone-free rate except for the difference between URSL and ESWL (p >0.05). For auxiliary procedures, the SUCRA rank was the same as final stone-free rate ( Figure-4 ).

**Figure 5:**
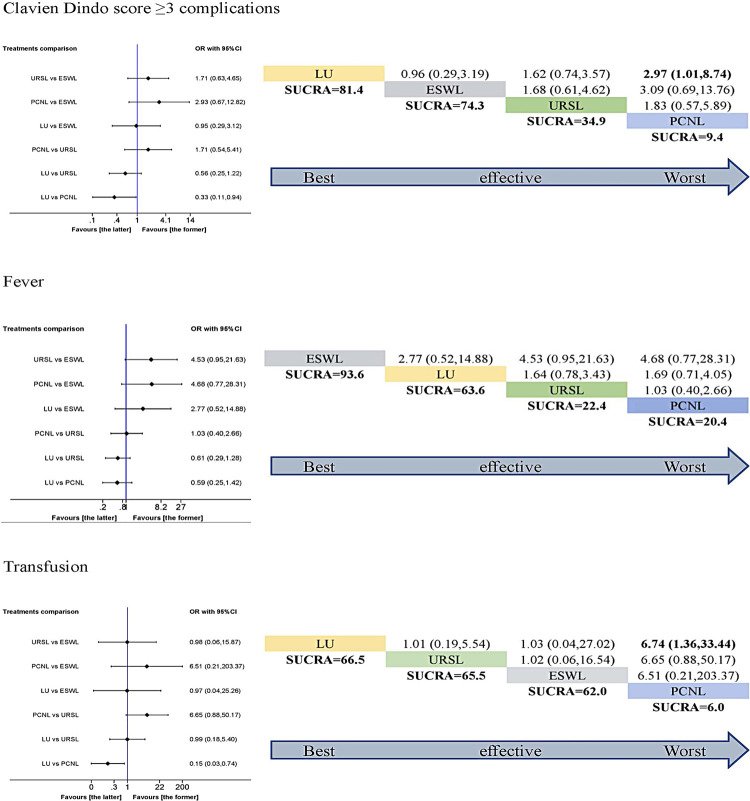
Pairwise meta-analysis (left) and SUCRA rank (right) in terms of Clavien Dindo score ≥ 3 complications, fever and transfusion. If the 95% CI was above or under 1.00, the difference was statistically significant (P< 0.05).

Both LU and PCNL were more effective for the initial stone-free rate, final stone-free rate and auxiliary procedures. However, the adverse events should be considered before making a decision. For Clavien Dindo score ≥3 complications, LU had the highest SUCRA score, followed by ESWL. While, PCNL had the lowest SUCRA score. LU was more effective than PCNL (p <0.05). The SUCRA outcome for Clavien Dindo score ≥3 complications indicated the following ranking: LU> ESWL> URSL> PCNL. For fever, ESWL had the highest SUCRA score, followed by LU. URSL and PCNL had almost the same SUCRA score. However, the difference did not reach statistical significance (p >0.05). The SUCRA ranking was as following: ESWL> LU> URSL> PCNL. For transfusion, LU had the highest SUCRA score, followed by URSL and ESWL. While, PCNL had the lowest SUCRA score. LU was more effective than PCNL (p <0.05). The SUCRA ranking was: LU> URSL> ESWL> PCNL ( Figure-5 ).

**Figure 6:**
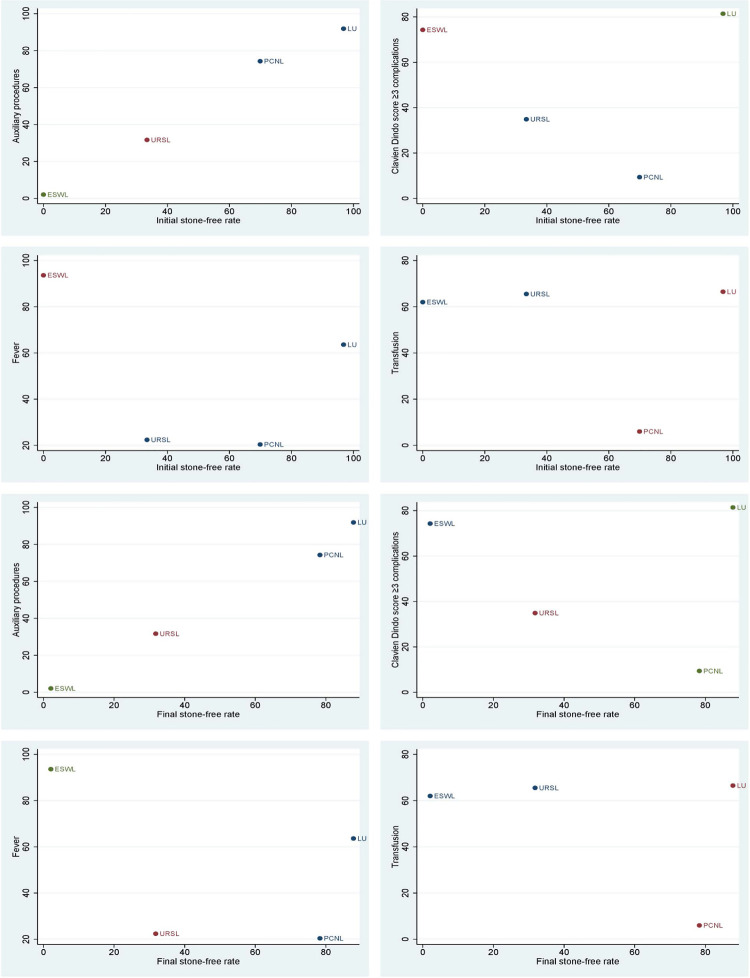
Cluster analysis for initial stone-free rate, final stone-free rate combined with auxiliary procedures, Clavien Dindo ≥ 3 complications, fever and transfusion.

Based on the results above, more effective surgical treatment may be associated with higher complications. How to choose the best treatment still needs further analysis. Cluster analysis results indicated that LU had the highest advantages and acceptable side effects. It is hard to evaluate the advantages of URSL and PCNL. However, considering the traumatic nature of PCNL, it should not be an option over URSL. ESWL had the lowest advantages for this situation ( Figure-6 ).

**Figure 7:**
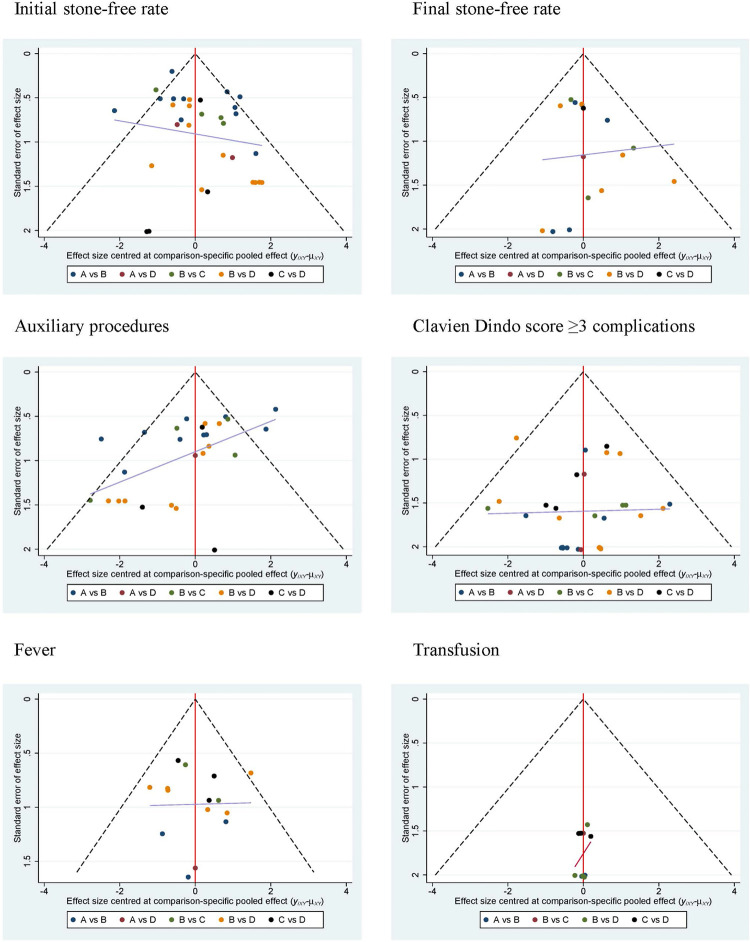
Network funnel plots to test the publication bias in terms of initial stone-free rate, final stone-free rate, auxiliary procedures, Clavien Dindo ≥ 3 complications, fever and transfusion.

There was little publication bias from funnel plots in each parameter ( Figure-7 ).

## DISCUSSION

In this network meta-analysis, three studies reported URSL-RIRS ( 27 , 30 , 34 ). Because some of the patients received URSL, other patients with stone retropulsion received RIRS. Two studies reported mPCNL ( 23 , 36 ). In addition, due to similar risk of complications and surgical outcomes, we combined mPCNL with PCNL and URSL-RIRS with URSL. This might have overestimated the efficacy of URSL and underestimated the efficacy of PCNL.

The primary outcomes of efficacy were initial and final stone-free rate. According to our results, LU showed the best initial and final stone-free rates with minimal auxiliary procedures, indicating its high efficacy. Based on the SUCRA rank, PCNL showed the second best initial and final stone-free rates. However, the difference between LU and PCNL did not reach statistical significance. Although, we overestimated the efficacy of URSL and underestimated the efficacy of PCNL by combining PCNL with mPCNL, URSL-RIRS with URSL. The efficacy of PCNL was still higher than URSL. While, URSL were significantly better than ESWL for initial stone-free rate. However, after more auxiliary procedures for ESWL, there was no significant difference in final stone-free rate between URSL and ESWL. The reason could be that the auxiliary procedures included URSL after initial failed ESWL. These results were consistent with reports of many other researchers. Yasui et al. reported high efficacy of stone-free rate for large proximal ureteral stones ( 9 ). Gaur et al. reported that LU had higher stone-free rate and less complications ( 37 ). Torricelli et al. performed a meta-analysis showing that LU was better than URSL ( 5 ). Although PCNL was not commonly used to treat the proximal ureteral stones. The efficacy of stone-free rate was almost as high as LU. Wang et al. reported LU and PCNL were more suitable for proximal ureteral impacted stones larger than 15mm ( 36 ). The AUA and EAU guidelines recommend URSL and ESWL as first-line options for ureteral stones less than 2cm ( 6 , 38 ). However, for the large proximal ureteral stones, the stone-free rate reported was 35-87% by URSL and 42% by ESWL ( 39 , 40 ). These results could not meet the clinical requirements.

Regarding adverse events, the most common complications are pain, fever, urine leakage, urinary tract infection and blood transfusions. Although LU is a more invasive procedure than ESWL, the risk of Clavien Dindo score ≥3 complications are similar. While, PCNL was associated with the worst Clavien Dindo score ≥3 complications, reflecting the high risk of PCNL. It might be the high fluid pressure during surgery, resulting in high fever rate of PCNL and URSL. Despite more invasive of LU and URSL, the transfusion rates of LU, URSL and ESWL were similar. However, PCNL had a significantly higher transfusion rate than LU and URSL.

Based on current studies, various surgical treatments have their own advantages and disadvantages. Although LU has a higher stone-free rate and fewer complications. It requires higher surgical techniques. PCNL has a similar efficacy as LU, but it could result in major complications and severe bleeding. The efficacy of URSL is lower than that of LU and PCNL, but the minimally invasive nature of URSL leads to better tolerance. Considering the efficacy and safety, Cluster analysis was applied in our study to evaluate the proper rank. LU had the highest initial and final stone-free rates and acceptable side effects. PCNL had higher initial and final stone-free rate than URSL. But the complications were more common in PCNL. It is difficult to draw a conclusion. However, considering the trauma of PCNL, we believe that it should not be an option over URSL. ESWL had the lowest advantages for the large proximal ureteral stones.

This study has a number of inherent limitations. First, the retrospective nature limited the quality of the results. Second, there were not enough studies to evaluate URSL-RIRS and mPCNL. The combination of mPCNL and PCNL, URSL-RIRS and URSL could lead to heterogeneities. Third, residual fragments were assessed by KUB or CT scan, which might have resulted in bias. Fourth, we didn’t evaluate ureteral stricture for a long-term follow-up. Fifth, we did not compare the transperitoneal or retroperitoneal LU. However, Singh et al. reported that there were no significant difference between transperitoneal LU and retroperitoneal LU ( 41 ). Sixth, the surgical costs were not available. Despite these deficiencies, this study will still help urologists select appropriate surgical treatments for large proximal ureteral stones.

## CONCLUSIONS

This network meta-analysis demonstrated that LU and PCNL had a higher efficacy on stone-free rate and auxiliary procedures for patients with proximal ureteral stones ≥10mm. PCNL could cause more serious complications. Therefore, LU have the potential to be considered as the first treatment choice of proximal ureteral stone ≥10mm.

## Figures and Tables

**Figure:**
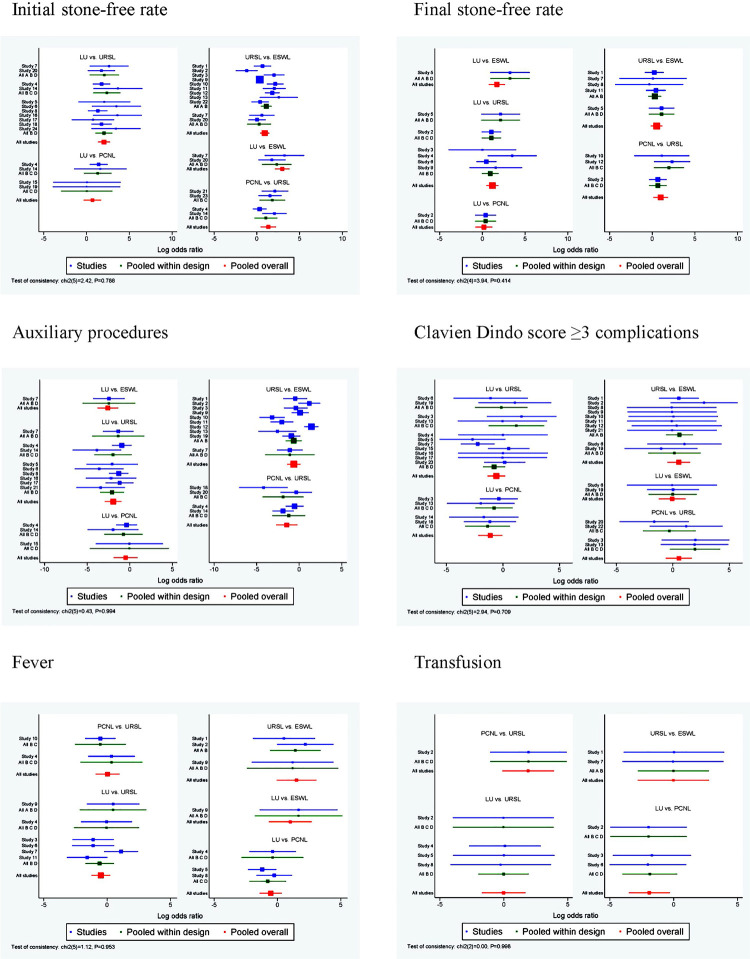
Network forest plots to test the consistency in terms of initial stone-free rate, final stone-free rate, auxiliary procedures, Clavien Dindo ≥ 3 complications, fever and transfusion. The difference was statistically significant if p was less than 0.05. A = ESWL; B = URSL; C = PCNL; D = LU.

**Figure:**
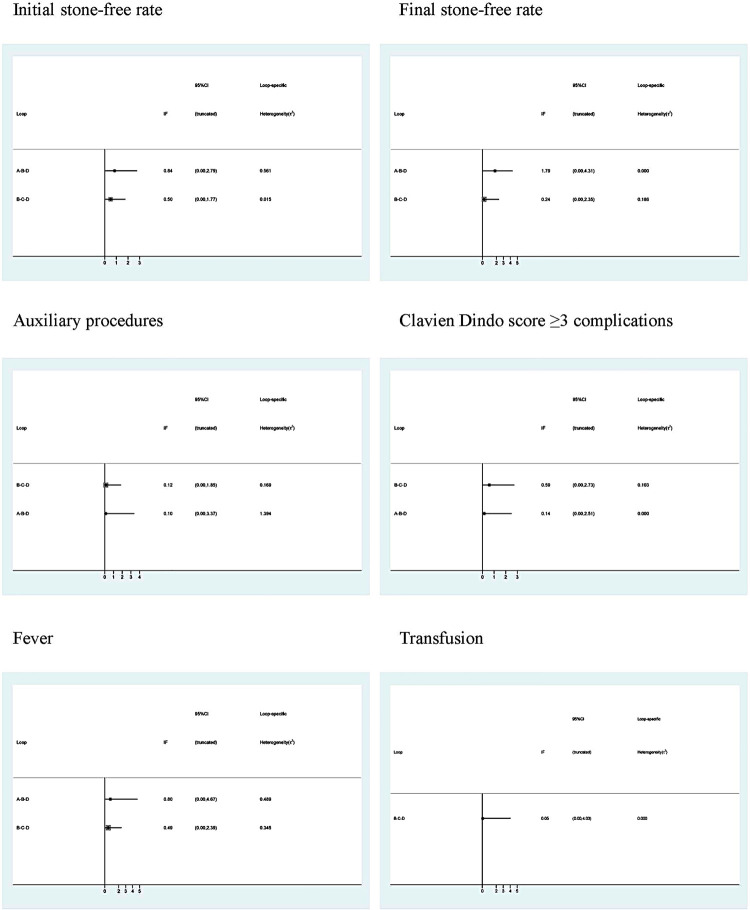
Loop consistency test in terms of initial stone-free rate, final stone-free rate, auxiliary procedures, Clavien Dindo >=3 complications, fever and transfusion. IF the 95% CI. Included 0, the difference was not statistically significant. A = ESWL; B = URSL; C = PCNL; D = LU.

## ABBREVIATIONS

LU: laparoscopic ureterolithotomy
PCNL: percutaneous nephrolithotomy
mPCNL: mini-percutaneous nephrolithotomy
ESWL: extracorporeal shock wave lithotripsy
URSL: ureteroscopic lithotripsy
URSL-RIRS: ureteroscopic lithotripsy-retrograde intrarenal surgery
SUCRA: surface under the cumulative ranking

## APPENDIX

**Appendix Box. Terminology: reviews with networks of multiple treatments**

**Different terms have been used to identify systematic reviews that incorporate a network of multiple treatment comparisons. A brief overview of common terms follows.**

Indirect treatment comparison: Comparison of 2 interventions for which studies against a common comparator, such as placebo or a standard treatment, are available (i.e., indirect information). The direct treatment effects of each intervention against the common comparator (i.e., treatment effects from a comparison of interventions made within a study) may be used to estimate an indirect treatment comparison between the 2 interventions ( Appendix Figure-1A ). An indirect treatment comparison (ITC) may also involve multiple links. For example, in Appendix Figure-1B , treatments B and D may be compared indirectly on the basis of studies encompassing comparisons of B versus C, A versus C, and A versus D.

Network meta-analysis or mixed treatment comparison: These terms, which are often used interchangeably, refer to situations involving the simultaneous comparison of 3 or more interventions. Any network of treatments consisting of strictly unclosed loops can be thought of as a series of ITCs ( Appendix Figures 1A and B ). In mixed treatment comparisons, both direct and indirect information is available to inform the effect size estimates for at least some of the comparisons; visually, this is shown by closed loops in a network graph ( Appendix Figure-1C ). Closed loops are not required to be present for every comparison under study. “Network meta-analysis” is an inclusive term that incorporates the scenarios of both indirect and mixed treatment comparisons.

Network geometry evaluation: The description of characteristics of the network of interventions, which may include use of numerical summary statistics. This does not involve quantitative synthesis to compare treatments. This evaluation describes the current evidence available for the competing interventions to identify gaps and potential bias. Network geometry is described further in **Appendix Box 4.**

**Appendix Box 1. The Assumption of Transitivity for Network Meta-Analysis**

Methods for indirect treatment comparisons and network meta-analysis enable learning about the relative treatment effects of, for example, treatments A and B through use of studies where these interventions are compared against a common therapy, C.

When planning a network meta-analysis, it is important to assess patient and study characteristics across the studies that compare pairs of treatments. These characteristics are commonly referred to as effect modifiers and include traits such as average patient age, gender distribution, disease severity, and a wide range of other plausible features.

For network meta-analysis to produce valid results, it is important that the distribution of effect modifiers is similar, for example, across studies of A versus B and A versus C. This balance increases the plausibility of reliable findings from an indirect comparison of B versus C through the common comparator A. When this balance is present, the assumption of transitivity can be judged to hold.

Authors of network meta-analyses should present systematic (and even tabulated) information regarding patient and study characteristics whenever available. This information helps readers to empirically evaluate the validity of the assumption of transitivity by reviewing the distribution of potential effect modifiers across trials.

**Appendix Box 2. Differences in Approach to Fitting Network Meta-Analyses**

Network meta-analysis can be performed within either a frequentist or a Bayesian framework. Frequentist and Bayesian approaches to statistics differ in their definitions of probability. Thus far, the majority of published network meta-analyses have used a Bayesian approach.

Bayesian analyses return the posterior probability distribution of all the model parameters given the data and prior beliefs (e.g., from external information) about the values of the parameters. They fully encapsulate the uncertainty in the parameter of interest and thus can make direct probability statements about these parameters (e.g., the probability that one intervention is superior to another).

Frequentist analyses calculate the probability that the observed data would have occurred under their sampling distribution for hypothesized values of the parameters. This approach to parameter estimation is more indirect than the Bayesian approach.

Bayesian methods have been criticized for their perceived complexity and the potential for subjectivity to be introduced by choice of a prior distribution that may affect study findings. Others argue that explicit use of a prior distribution makes transparent how individuals can interpret the same data differently. Despite these challenges, Bayesian methods offer considerable flexibility for statistical modeling.

In-depth introductions to Bayesian methods and discussion of these and other issues can be found elsewhere.

**Appendix Box 3. Network Meta-Analysis and Assessment of Consistency**

Network meta-analysis often involves the combination of direct and indirect evidence. In the simplest case, we wish to compare treatments A and B and have 2 sources of information: direct evidence via studies comparing A versus B, and indirect evidence via groups of studies comparing A and B with a common intervention, C. Together, this evidence forms a closed loop, ABC.

Direct and indirect evidence for a comparison of interventions should be combined only when their findings are similar in magnitude and interpretation. For example, for a comparison of mortality rates between A and B, an odds ratio determined from studies of A versus B should be similar to the odds ratio comparing A versus B estimated indirectly based on studies of A versus C and B versus C. This assumption of comparability of direct and indirect evidence is referred to as consistency of treatment effects.

When a treatment network contains a closed loop of interventions, it is possible to examine statistically whether there is agreement between the direct and indirect estimates of intervention effect.

Different methods to evaluate potential differences in relative treatment effects estimated by direct and indirect comparisons are grouped as local approaches and global approaches. Local approaches (e.g., the Bucher method or the node-splitting method) assess the presence of inconsistency for a particular pairwise comparison in the network, whereas global approaches (e.g., inconsistency models, I2 measure for inconsistency) consider the potential for inconsistency in the network as a whole.

Tests for inconsistency can have limited power to detect a true difference between direct and indirect evidence. When multiple loops are being tested for inconsistency, one or a few may show inconsistency simply by chance. Further discussions of consistency and related concepts are available elsewhere.

Inconsistency in a treatment network can indicate lack of transitivity ( **see Appendix Box 1** ).

**Appendix Box 4. Network Geometry and Considerations for Bias**

The term network geometry is used to refer to the architecture of the treatment comparisons that have been made for the condition under study. This includes what treatments are involved in the comparisons in a network, in what abundance they are present, the respective numbers of patients randomly assigned to each treatment, and whether particular treatments and comparisons may have been preferred or avoided.

Networks may take on different shapes. Poorly connected networks depend extensively on indirect comparisons. Meta-analyses of such networks may be less reliable than those from networks where most treatments have been compared against each other.

Qualitative description of network geometry should be provided and accompanied by a network graph. Quantitative metrics assessing features of network geometry, such as diversity (related to the number of treatments assessed and the balance of evidence among them), co-occurrence (related to whether comparisons between certain treatments are more or less common), and homophily (related to the extent of comparisons between treatments in the same class versus competing classes), can also be mentioned.

Although common, established steps for reviewing network geometry do not yet exist, however examples of in-depth evaluations have been described related to treatments for tropical diseases and basal cell carcinoma and may be of interest to readers. An example based on 75 trials of treatments for pulmonary arterial hypertension ( Appendix Figure-3 ) suggests that head-to-head studies of active therapies may prove useful to further strengthen confidence in interpretation of summary estimates of treatment comparisons.

**Appendix Box 5. Probabilities and Rankings in Network Meta-Analysis**

Systematic reviews incorporating network meta-analyses can provide information about the hierarchy of competing interventions in terms of treatment rankings.

The term treatment ranking probabilities refers to the probabilities estimated for each treatment in a network of achieving a particular placement in an ordering of treatment effects from best to worst. A network of 10 treatments provides a total of 100 ranking probabilities—that is, for each intervention, the chance of being ranked first, second, third, fourth, fifth, and so forth).

Several techniques are feasible to summarize relative rankings, and include graphical tools as well as different approaches for estimating ranking probabilities. Appendix Figure-6 shows 2 approaches to presenting such information, on the basis of a comparison of adjuvant interventions for resected pancreatic adenocarcinoma.

Robust reporting of rankings also includes specifying median ranks with uncertainty intervals, cumulative probability curves, and the surface under the cumulative ranking (SUCRA) curve.

Rankings can be reported along with corresponding estimates of pairwise comparisons between interventions. Rankings should be reported with probability estimates to minimize misinterpretation from focusing too much on the most likely rank.

Rankings may exaggerate small differences in relative effects, especially if they are based on limited information. An objective assessment of the strength of information in the network and the magnitude of absolute benefits should accompany rankings to minimize potential biases.

## Supplementary Table 1

**Table:** PRISMA NMA Checklist of Items to Include When Reporting A Systematic Review Involving a Network Meta-analysis.

Section/Topic	Item #	Checklist Item	Reported on Page #
**TITLE**			
Title	1	Identify the report as a systematic review *incorporating a network meta-analysis (or related form of meta-analysis).*	1
**ABSTRACT**			
Structured summary	2	Provide a structured summary including, as applicable: **Background:** main objectives **Methods:** data sources; study eligibility criteria, participants, and interventions; study appraisal; and *synthesis methods, such as network meta-analysis.* **Results:** number of studies and participants identified; summary estimates with corresponding confidence/credible intervals; *treatment rankings may also be discussed. Authors may choose to summarize pairwise comparisons against a chosen treatment included in their analyses for brevity.* **Discussion/Conclusions:** limitations; conclusions and implications of findings. Other: primary source of funding; systematic review registration number with registry name.	2
**INTRODUCTION**			
Rationale	3	Describe the rationale for the review in the context of what is already known, *including mention of why a network meta-analysis has been conducted.*	3
Objectives	4	Provide an explicit statement of questions being addressed, with reference to participants, interventions, comparisons, outcomes, and study design (PICOS).	3, 4
**METHODS**			
Protocol and registration	5	Indicate whether a review protocol exists and if and where it can be accessed (e.g., Web address); and, if available, provide registration information, including registration number.	5
Eligibility criteria	6	Specify study characteristics (e.g., PICOS, length of follow-up) and report characteristics (e.g., years considered, language, publication status) used as criteria for eligibility, giving rationale. *Clearly describe eligible treatments included in the treatment network, and note whether any have been clustered or merged into the same node (with justification).*	5, 6
Information sources	7	Describe all information sources (e.g., databases with dates of coverage, contact with study authors to identify additional studies) in the search and date last searched.	5
Search	8	Present full electronic search strategy for at least one database, including any limits used, such that it could be repeated.	5
Study selection	9	State the process for selecting studies (i.e., screening, eligibility, included in systematic review, and, if applicable, included in the meta-analysis).	5, Fig 1
Data collection process	10	Describe method of data extraction from reports (e.g., piloted forms, independently, in duplicate) and any processes for obtaining and confirming data from investigators.	5, 6
Data items	11	List and define all variables for which data were sought (e.g., PICOS, funding sources) and any assumptions and simplifications made.	6
**Geometry of the network**	S1	Describe methods used to explore the geometry of the treatment network under study and potential biases related to it. This should include how the evidence base has been graphically summarized for presentation, and what characteristics were compiled and used to describe the evidence base to readers.	6, Table-1
Risk of bias within individual studies	12	Describe methods used for assessing risk of bias of individual studies (including specification of whether this was done at the study or outcome level), and how this information is to be used in any data synthesis.	6, 7, Figure-2
Summary measures	13	State the principal summary measures (e.g., risk ratio, difference in means). *Also describe the use of additional summary measures assessed, such as treatment rankings and surface under the cumulative ranking curve (SUCRA) values, as well as modified approaches used to present summary findings from meta-analyses.*	7
Planned methods of analysis	14	Describe the methods of handling data and combining results of studies for each network meta-analysis. This should include, but not be limited to: Handling of multi-arm trials; Selection of variance structure; Selection of prior distributions in Bayesian analyses; and Assessment of model fit.	7
**Assessment of Inconsistency**	S2	Describe the statistical methods used to evaluate the agreement of direct and indirect evidence in the treatment network(s) studied. Describe efforts taken to address its presence when found.	7
Risk of bias across studies	15	Specify any assessment of risk of bias that may affect the cumulative evidence (e.g., publication bias, selective reporting within studies).	6, 7
Additional analyses	16	Describe methods of additional analyses if done, indicating which were pre-specified. This may include, but not be limited to, the following: Sensitivity or subgroup analyses; Meta- *regression analyses;* Alternative formulations of the treatment network; and Use of alternative prior distributions for Bayesian analyses (if applicable).	7
**RESULTS†**			
Study selection	17	Give numbers of studies screened, assessed for eligibility, and included in the review, with reasons for exclusions at each stage, ideally with a flow diagram.	8, Fig 1
**Presentation of network structure**	S3	Provide a network graph of the included studies to enable visualization of the geometry of the treatment network.	8, and Fig 3
**Summary of network geometry**	S4	Provide a brief overview of characteristics of the treatment network. This may include commentary on the abundance of trials and randomized patients for the different interventions and pairwise comparisons in the network, gaps of evidence in the treatment network, and potential biases reflected by the network structure.	8, 9, 10, Fig 2, Table-2
Study characteristics	18	For each study, present characteristics for which data were extracted (e.g., study size, PICOS, follow-up period) and provide the citations.	8, 9, 10, and Table-1
Risk of bias within studies	19	Present data on risk of bias of each study and, if available, any outcome level assessment.	Figure-2
Results of individual studies	20	For all outcomes considered (benefits or harms), present, for each study: 1) simple summary data for each intervention group, and 2) effect estimates and confidence intervals. *Modified approaches may be needed to deal with information from larger networks.*	8, 9, 10, Figure-4, Figure-5, and Figure-6
Synthesis of results	21	Present results of each meta-analysis done, including confidence/credible intervals. *In larger networks, authors may focus on comparisons versus a particular comparator (e.g. placebo or standard care), with full findings presented in an appendix. League tables and forest plots may be considered to summarize pairwise comparisons* . If additional summary measures were explored (such as treatment rankings), these should also be presented.	8, 9, 10, Table-2
**Exploration for inconsistency**	S5	Describe results from investigations of inconsistency. This may include such information as measures of model fit to compare consistency and inconsistency models, P values from statistical tests, or summary of inconsistency estimates from different parts of the treatment network.	8, 9, Suppl. Figure-1, and Suppl. Figure-2
Risk of bias across studies	22	Present results of any assessment of risk of bias across studies for the evidence base being studied.	8, 9, and Figure-2
Results of additional analyses	23	Give results of additional analyses, if done (e.g., sensitivity or subgroup analyses, meta-regression analyses, *alternative network geometries studied, alternative choice of prior distributions for Bayesian analyses, and so forth).*	10, and Figure-7
**DISCUSSION**			
Summary of evidence	24	Summarize the main findings, including the strength of evidence for each main outcome; consider their relevance to key groups (e.g., healthcare providers, users, and policy-makers).	11, 12, 13
Limitations	25	Discuss limitations at study and outcome level (e.g., risk of bias), and at review level (e.g., incomplete retrieval of identified research, reporting bias). *Comment on the validity of the assumptions, such as transitivity and consistency. Comment on any concerns regarding network geometry (e.g., avoidance of certain comparisons).*	13
Conclusions	26	Provide a general interpretation of the results in the context of other evidence, and implications for future research.	14
**FUNDING**			
Funding	27	Describe sources of funding for the systematic review and other support (e.g., supply of data); role of funders for the systematic review. This should also include information regarding whether funding has been received from manufacturers of treatments in the network and/or whether some of the authors are content experts with professional conflicts of interest that could affect use of treatments in the network.	None

**PICOS** = population, intervention, comparators, outcomes, study design.

* Text in italics indicateS wording specific to reporting of network meta-analyses that has been added to guidance from the PRISMA statement.

† Authors may wish to plan for use of appendices to present all relevant information in full detail for items in this section.
